# Prevalence of Latent and Active Tuberculosis Infection in Inflammatory Bowel Disease Patients Who Received Biological Treatment

**DOI:** 10.5152/tjg.2026.25072

**Published:** 2026-02-13

**Authors:** Saida Dashdamirova, Tugce Eskazan, Yusuf Ziya Erzin, Aykut Ferhat Celik, Gülen Hatemi, Ali Ibrahim Hatemi

**Affiliations:** 1 Department of Internal Medicine, İstanbul University-Cerrahpaşa Cerrahpaşa Faculty of Medicine, İstanbul, Türkiye; 2 Division of Gastroenterology, Department of Internal Medicine, İstanbul University-Cerrahpaşa Cerrahpaşa Faculty of Medicine, İstanbul, Türkiye; 3 Division of Rheumatology, Department of Internal Medicine, İstanbul University-Cerrahpaşa Cerrahpaşa Faculty of Medicine, İstanbul, Türkiye

**Keywords:** Adalimumab, anti-TNF, inflammatory bowel disease, infliximab, isoniazide, latent tuberculosis, tuberculosis, vedolizumab

## Abstract

**Background/Aims:**

This study aims to assess the frequency of latent tuberculosis (TB) and the incidence of active TB infection in patients with inflammatory bowel disease (IBD) and Behçet’s syndrome with gastrointestinal involvement who received either tumor necrosis factor–alpha (TNF-α) antagonist therapy or vedolizumab as a second-line biologic treatment following TNF-α antagonist therapy.

**Materials and Methods:**

A total of 349 patients were included in the study. Demographic data, disease characteristics, TB screening results, prophylactic treatment regimens and the duration, as well as side effects were collected from patient records.

**Results:**

Among the patients, 196 (56.1%) were male, with a mean age of 42 years and a mean disease duration of 10 ± 6 years. A total of 267 (76.5%) had Crohn’s disease, 76 (21.8%) had ulcerative colitis, 3 had indeterminate colitis (0.8%), and 3 had Behçet’s syndrome with gastrointestinal involvement (0.8%). Latent TB was diagnosed in 176 (50.4%) patients, and 162 (92%) of them received isoniazid prophylaxis. Six (1.7%) patients developed active TB infection during the treatment period, occurring between 2 and 48 months after initiating TNF-α antagonist therapy. No cases of tuberculosis were observed in patients who had vedolizumab treatment. Among those who developed TB, 3 patients had pulmonary TB, and 3 had both pulmonary and extrapulmonary TB. No significant association was found between TB development and IBD phenotype or isoniazid use.

**Conclusion:**

Despite latent TB screening and prophylactic treatment, patients receiving TNF-α antagonists remain at risk of developing active TB, with a latent TB prevalence of 50.4% and an active TB incidence of 1.7%. These findings underscore the continued need for vigilance and robust TB monitoring strategies in this patient population.

**Main Points**

- In this cohort of 349 inflammatory bowel disease patients receiving anti-tumor necrosis factor therapy, the prevalence of latent tuberculosis infection was 50.4%, and 92% of these patients received isoniazid prophylaxis.
- The positivity rates for the tuberculin skin test and Quantiferon test were 54.7% and 20.9%, respectively.
- Despite appropriate screening and prophylaxis, the incidence of active tuberculosis was 1.7% (6 out of 349 patients).
- Among the 6 patients with active tuberculosis, three had pulmonary involvement, while the other three had both pulmonary and extrapulmonary disease.

## Introduction

In the current management of inflammatory bowel disease (IBD), both biologic agents and small molecules are widely employed.^1^^,^^2^ A study conducted in Türkiye reported that approximately 36% of patients with IBD were treated with tumor necrosis factor–alpha (TNF-α) inhibitors.^1^

Tumor necrosis factor–α plays a crucial role in the formation that limits mycobacterial spread by binding to *TNFR1*.^3^^-^^5^ Inhibition of TNF-α disrupts this defense mechanism, facilitating Mycobacterium tuberculosis proliferation and increasing the risk of tuberculosis (TB).

Latent tuberculosis infection (LTBI) occurs when Mycobacterium tuberculosis induces an immune response without active TB symptoms. The World Health Organization (WHO) recommends LTBI screening prior to the initiation of anti-TNF therapy.^6^ Similarly, the Turkish Ministry of Health’s guidelines also advise screening for LTBI using tuberculin skin tests (TSTs) or interferon gamma release assays (IGRAs) before initiating anti-TNF treatment. In cases of positive screening results, prophylactic treatment for LTBI should be administered.^7^ Continued monitoring for TB is essential during anti-TNF therapy, and annual screening is recommended for patients who initially test negative. If the TST result is negative, a booster TST or IGRAs should be performed. Preventive treatment is indicated for positive IGRA or induration greater than 5 mm on TST.^8^ It is estimated that approximately 23% of the global population (about 1.7 billion people) have LTBI.^9^ BCG (Bacillus Calmette–Guérin) vaccination can lead to false-positive TST results, and the use of prednisolone or azathioprine may result in false-negative TST results.^10^

This retrospective study aimed to determine the frequency of LTBI in patients eligible for anti-TNF treatment, evaluate the incidence of active TB infection during treatment with anti-TNF agents and second-line vedolizumab therapies, identify predictors of active TB, and assess the efficacy and safety of isoniazid (INH) prophylaxis in this patient cohort.

## Materials and Methods

This retrospective study included IBD patients and Behçet’s syndrome patients with gastrointestinal involvement who received anti-TNF or second-line vedolizumab therapy at Cerrahpasa Faculty of Medicine Gastroenterology Outpatient Clinic between 1998 and 2018. Data were collected from patient files and the hospital’s electronic medical records. The following data were recorded: age, gender, disease characteristics, age at IBD diagnosis, duration of follow-up, previous and current medical treatments, comorbidities, and results of latent TB screening tests. For patients who received prophylactic treatment, data on the duration and side effects of INH prophylaxis were documented. Information on patients who developed active tuberculosis infection was also recorded and analyzed with a focus on clinical features and treatment outcomes.

**Ethical Approval**

The study was approved by the local ethics committee of İstanbul University-Cerrahpaşa, Cerrahpaşa Faculty of Medicine (Decision No: 62411 dated: 24.09.2018). All procedures performed in studies involving human participants were in accordance with ethical standards of the institutional and/or national research committee and with the 1964 Helsinki declaration and its later amendments or comparable ethical standards. Due to the retrospective nature of the study, the requirement for written informed consent was waived by the local ethics committee.

### Statistical Analysis

All analyses were performed using IBM SPSS Statistics for Windows, Version 26.0 (IBM SPSS Corp.; Armonk, NY, USA). Categorical variables are expressed as percentages, and continuous variables as mean ± SD for normally distributed data, or as median with interquartile range for non-normally distributed data. The Pearson chi-square test was used for categorical variable comparison, and the Mann–Whitney *U*-test for non-normally distributed continuous variables. *P*-value <.05 was considered statistically significant at a 95% CI.

## Results

### Demographic Features

A total of 349 patients were included in the study. The demographic and clinical characteristics are shown in Table 1. Among them, 196 (56.1%) were male, 153 (43.8%) were female, with a mean age of 42 ± 14 years and a mean disease duration of 10 ± 6 years. The diagnosis was Crohn’s disease (CD) in 267 (76.5%), ulcerative colitis (UC) in 76 (21.8%), indeterminate colitis in 3 (0.8%), and Behçet’s syndrome with gastrointestinal involvement in 3 (0.8%).

A total of 77% (n = 269) of patients were treated with infliximab, 48.1% (n = 168) with adalimumab, 5.7% (n = 20) with certolizumab, 5.4% (n = 19) with vedolizumab, and 1.1% (n = 4) with golimumab. Owing to reimbursement policies at the time of the study, vedolizumab and golimumab were not initiated as first-line therapies. Consequently, all patients treated with these agents had a prior history of infliximab and/or adalimumab use.

In CD patients, disease locations included ileocolonic (L3) in 60.7%, ileal (L1) in 21.3%, and colonic in 14.2%. In UC patients, 46.1% had pancolitis (E3), 39.5% had left-sided colitis (E2), and 13.2% had extensive colitis.

### History of Past Tuberculosis Infection and Exposure to an Infected Person

A total of 8 patients had a history of prior TB infection. All were treated with the standard 6-month regimen, including the intensive phase with 4 drugs (INH, rifampin, pyrazinamide, and ethambutol) for 2 months, followed by the continuation phase with INH and rifampin for at least 4 more months. None of these patients experienced TB reactivation during anti-TNF therapy.

Fourteen patients had close contact with individuals diagnosed with active pulmonary TB prior to initiating anti-TNF therapy, of whom 9 received INH prophylaxis. Despite INH treatment, 1 patient developed active TB during the third month of anti-TNF therapy. This patient had previously completed a 6-month course of INH due to her husband’s pulmonary TB diagnosis. Among the 5 patients with prior TB exposure who did not receive INH, the QuantiFERON-TB Gold (QFT) test was negative; however, one of these patients developed active TB. Notably, this patient’s exposure occurred over 10 years prior, and his TST induration was also negative.

### Results of Screening Tests for for Latent Tuberculosis Infection

**Figure 1.**
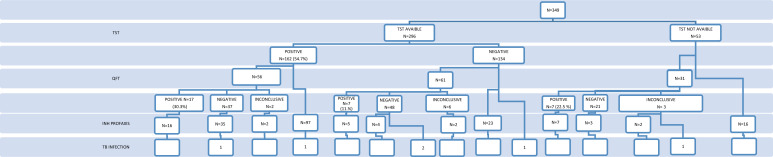
Flowchart of latent tuberculosis screening results and isoniazid prophylaxis in patients receiving anti-TNF therapy. TST, tuberculin skin test.

Among the 349 patients, 296 (84.8%) underwent a TST, of whom 162 (54.7%) had an induration of ≥5 mm. Among these 162 TST-positive patients, 56 also underwent QFT testing, with 17 (30%) yielding positive results (Table 2 and Figure 1). The immunosuppressive status of patients at the time of tuberculosis screening can be summarizedas follows: 3 patients had negative TST results, 2 of whom were receiving azathioprine, while 1 was receiving both azathioprine and corticosteroids. One patient with an unavailable TST result had an indeterminate QFT test and was under corticosteroid therapy (Table 3).

### Isoniazid Prophylaxis

Among 162 patients who had TST positivity, 150 received prophylaxis (92.5%). Among 31 patients who had QFT positivity, 28 received prophylaxis (90.3%). Data about INH prophylaxis duration were available in 159 patients; 27 patients completed the INH treatment in less than 9 months (16.9%); 132 patients (83%) completed the 9-month prophylaxis. Among the entire group, the INH prophylaxis rate among patients with latent TB infection was 92% (162/176) (Figure 1).

### Isoniazid-Related Adverse Events

Ten patients (4.7%) experienced adverse events, with the most common being elevated liver enzymes (n = 7, 3.3%). One patient developed a rash, 1 had neuropathy, and 1 developed myositis. Among those with adverse events, 40% completed treatment within 9 months.

### Patients Who Developed Active Tuberculosis During Anti-Tumor Necrosis Factor Treatment

Tuberculosis infection developed in 6 patients (1.7%) (Tables 3 and 4). Two patients who had used INH for 6 and 9 months, both on azathioprine and anti-TNF therapy, developed TB. One patient, who had received 6 months of INH before anti-TNF therapy due to her husband’s TB, developed TB in the third month of anti-TNF treatment. The other, who received 9 months of INH, developed TB after 48 months of TNF-α antagonist therapy, suggesting a new infection. Four patients who developed TB had not received INH. Two of them had negative TST and QFT tests, with active TB developing 7 and 44 months after starting anti-TNF therapy. In the other 2, latent TB screening was insufficient: 1 had a 0-mm TST wheal and no booster dose or QFT test, developing TB 7 months after anti-TNF therapy; the other had an indeterminate QFT result and no TST, developing TB 2 months after starting anti-TNF therapy. All 6 patients with TB had lung involvement, with 1 also having spleen and peritoneum involvement, 1 with concurrent lymph node involvement, and 1 with spleen involvement (Table 4). Five of the 6 resumed anti-TNF therapy after completing TB treatment, with intervals of 4.5 years, 4 years, 3.5 years, 5 months, and 3 months. No significant differences were observed between those who developed TB and those who did not in clinical parameters (Table 5).

## Discussion

In this study, the prevalence of latent and active TB infections in IBD patients on TNF-alpha antagonist therapy was assessed. In Türkiye, TB incidence was 14.6 per 100 000 in 2017.^10^ In this study, 50.4% of IBD patients had latent TB (176/349), and 6 patients (1.7%) developed active TB after a median of 30 months of TNF-α antagonist therapy. Keane et al^11^ reported that 0.05% (70 out of 147 000) of patients on infliximab between 1998 and 2001 developed TB; similarly, Hanta et al^12^ found TB in 1.5% of 179 patients treated with TNF-α antagonists over 3 years. In Türkiye, studies have also documented TB incidence in patients on TNF-α antagonists, including 6 cases (0.85%) in 702 patients,^13^ 2 (1.1%) in 179 patients,^14^ and 3 (3.9%) in 76 IBD patients.^15^ Borekci’s 2015 study of 1964 patients found 16 cases of TB, with an incidence 21 times higher than the general population.^16^ Similar findings have been reported in other countries as well. In a 2015 study, Kim et al^17^ reported 4.2% of TB infection in 376 IBD patients, and Byun reported 3.1% of TB infection in 160 patients receiving TNF-α antagonist therapy.^18^

Since TNF-α antagonist therapy increases the risk of TB, it is recommended that all patients be screened for LTBI before starting treatment. Prophylactic TB therapy should be administered to those with a positive LTBI result. While there is no gold standard test for LTBI screening, the TST and IGRAs are commonly used. Studies have shown that neither TST nor IGRAs are superior in predicting active TB disease when used for LTBI screening. The tuberculin skin test has high specificity for detecting LTBI in populations without the BCG vaccine (97%), but its specificity decreases in BCG-vaccinated populations. In contrast, IGRAs maintain high specificity and are unaffected by the BCG vaccine. Regarding sensitivity, different results have been reported, with the T-spot TB test showing higher sensitivity than both. According to the WHO’s 2018 recommendations,^6^ both tests are suitable for screening. The European Centre for Disease Prevention and Control recommends TST for patients with close contact to TB infection and IGRAs for immunosuppressed patients with negative TST results.^19^ Interferon gamma release assays are a more costly option that generally necessitates the use of a private laboratory in the country. However, it offers the advantage of providing results after a single visit and within a relatively short time frame. Conversely, TST is a more cost-effective option, albeit with a longer turnaround time for results, which are obtained during a second visit 72 hours after testing and require interpretation by an experienced healthcare professional. The TB Diagnosis and Treatment Guidelines advocate that TST should be the initial screening test in the country, with IGST (interferon-gamma release test) being the preferred option for individuals who test negative with TST after the booster dose or for those who will receive immunosuppressive therapy.^10^

In the study by Kim et al,^17^ the LTBI positivity rate among IBD patients was reported to be 8%, while another study found it to be 6.9%.^20^ Despite the relatively low positivity rates observed in these Korean and Spanish studies, it is important to note that Türkiye has a high endemic rate of TB, as evidenced by the higher positivity rates documented in studies conducted in Türkiye. For instance, in the study by Cekic et al,^15^ 56.2% of patients tested positive for LTBI. In another study, Dogan et al^14^ reported TST positivity in 127 (70.9%) of 179 patients. Cağatay et al^13^ also reported TST positivity at 61.8% in a 2009 study. In the current cohort, the LTBI positivity rate was 54.7% with TST and 20.9% with the QFT test. A previous study from the Rheumatology department of the hospital also showed high LTBI positivity, with 57.7% for TST and 30% for QFT.^8^ The high TST positivity rate detected in 2 different studies from the hospital is likely attributable to the routine TB vaccination given in childhood in the country. In the present study, among patients with a PPD (purified protein derivative) greater than 5 mm, 30% exhibited a positive QFT test result. It was observed that if the decision regarding INH prophylaxis, made solely based on TST results, is made, this will result in a more than 2-fold increase in the rate of unnecessary prophylaxis compared to that indicated by IGRAs tests.

Tuberculosis reactivation presents a potentially life-threatening condition and can manifest in extrapulmonary or disseminated forms in immunocompromised patients.^10^ In the present series, isolated pulmonary TB was observed in 3 cases, while pulmonary and extrapulmonary TB were noted in 3 other patients. According to the 2019 Report on TB in Türkiye, the frequency of pulmonary TB and extrapulmonary TB was reported to be 66.1% and 33.9%, respectively.^10^ Byun et al^18^ evaluated 873 IBD patients who had received TNF-α antagonist therapy, and TB developed in 25 of these patients, 84% of them being pulmonary TB. In a further study, Lee et al^21^ evaluated 821 patients, 10 of whom developed TB, with all patients exhibiting pulmonary involvement. In another study, 57% of 70 patients had extrapulmonary TB, and 31% had pulmonary TB.^11^ A study from Türkiye revealed that all patients with TB reactivation had extrapulmonary involvement, with 1 patient being diagnosed with disseminated TB.^22^

In the present study, TB was diagnosed after a mean of 18.5 months (median: 7 months) of anti-TNF treatment. TB developed in 4 (67%) of 6 patients within the first 7 months, suggesting that these patients are likely to experience TB reactivation. In 2 patients, TB developed 44 and 48 months after the initiation of anti-TNF therapy, indicating that these patients likely had a new TB infection. The findings of the present study are in close alignment with those of the aforementioned study. In the study by Cekic et al,^15^ TB infections developed in 1 of 3 patients within 1 year of initiating anti-TNF treatment. In another study from Türkiye, 4 patients who developed reactivation TB were diagnosed 18-84 months after the initiation of anti-TNF treatment.^22^ In a study by Lee et al,^21^ active TB was diagnosed in 3 out of 10 patients over a 1-year period. These findings suggest that TNF antagonist therapy may predispose to reactivation, as well as to the primary TB infection. In this series, none of the 8 patients with a prior TB infection experienced TB reactivation during TNF-α antagonist therapy. In a similar manner, Dogan et al^14^ found no evidence of TB infection in any patient who had been previously treated for TB and subsequently received TNF-α antagonist therapy. These findings provide support for the hypothesis that TNF-alpha antagonists can be safely used in patients with a history of TB infection, provided they have been appropriately treated.

In the present study, active TB developed exclusively in patients receiving infliximab and adalimumab, with no cases observed in those treated with certolizumab, golimumab, or vedolizumab. However, it should be noted that only 12.3% of patients were treated with biologics other than infliximab or adalimumab. Because of the reimbursement policies during the study period, none of the patients had vedolizumab as a first-line therapy. Of the 6 patients who subsequently developed TB, 3 were using infliximab, and 3 were using adalimumab. In the study by Borekci et al,^16^ 9 of 16 patients who developed TB were receiving infliximab, while 3 were on adalimumab. A similar observation was made in another study, where 9 of 10 patients were treated with infliximab.^17^ In the study by Byun et al,^18^ infliximab was used in all 5 patients. Some studies suggest that infliximab and adalimumab are associated with a higher risk of TB compared to etanercept.^23^ ^24^ Nevertheless, the present study did not identify any substantial disparities in the occurrence of TB infection among patients treated with different anti-TNF agents. Consistent with these findings, a study by Borekci et al^16^ failed to demonstrate a correlation between the development of TB infection and the specific TNF-α antagonist employed. In this study, the incidence of TB in patients receiving infliximab was 1.4%, which is analogous to the findings of Borekci et al^16^ (1.31%).

At the time of active TB diagnosis, 5 out of 6 patients (83.3%) were receiving azathioprine in addition to anti-TNF therapy, 1 patient (16.6%) was on both azathioprine and corticosteroids, and 1 patient was receiving anti-TNF monotherapy. Consistent with the findings of this study, a study by Kim et al^17^ revealed that the majority of patients (93.8%) were using an additional immunosuppressive agent (either steroids or azathioprine) during anti-TNF therapy when active TB developed, while only 1 patient was on anti-TNF monotherapy.

Actually, all patients in this cohort had prior exposure to azathioprine. Moreover, as noted above, the majority of patients continued to receive immunomodulators concomitantly during anti-TNF therapy. This combined immunosuppression represents an additional risk factor for tuberculosis reactivation, particularly in TB-endemic regions.^25^ This concern has also been highlighted by Fortes et al,^26^ who identified thiopurines as an independent risk factor for TB, with azathioprine alone increasing the risk by 5.8-fold and the combination of azathioprine with anti-TNF therapy associated with a 9-fold increase in TB risk. In this cohort, only 1 of the TB patients had concomitant corticosteroid use at the time of reactivation. However, one-third of patients had a history of corticosteroid exposure prior to initiating anti-TNF therapy. A meta-analysis reported that corticosteroid use may increase the risk of active tuberculosis by approximately 8-fold,^25^ underscoring the importance of considering cumulative immunosuppressive burden when evaluating TB risk in IBD patients receiving biologic therapy.

In the present study, 2 out of 6 patients who developed TB had received INH prophylaxis. These findings suggest that INH prophylaxis does not guarantee protection against TB infection. Indeed, INH prophylaxis has been shown to only prevent the reactivation of latent TB infections; de novo TB infections may still occur during long-term anti-TNF therapy. Consequently, periodic screening for LTBI should be considered during prolonged TNF antagonist therapy, particularly in countries with a high incidence of TB.

Despite its retrospective nature, this real-life dataset offers clinically meaningful insights. Firstly, although there is general awareness of TB risk in the country, these findings indicate gaps in screening practices. For example, among patients with a negative TST, only 45% (61/134) underwent QFT, despite guidelines recommending a booster dose of TST or IGRA testing in such cases. Moreover, LTBI screening was not appropriately conducted in 2 of the 6 patients who developed TB in this study. This underscores the potential for oversight of proper LTBI screening, even in a TB-endemic country. Secondly, INH prophylaxis may only prevent the reactivation of latent TB infection, meaning the risk of de novo TB infection remains present during long-term anti-TNF therapy. Two patients who developed active TB infection long after initiating anti-TNF treatment (44 and 48 months) were identified, suggesting the possibility of new infection rather than reactivation of latent TB. Thirdly, an IGRA test is more appropriate for screening in Türkiye due to the high false positivity rate of TST due to BCG immunization. While 54.7% (162/296) of the cohort had a positive TST, only 20.9% (31/148) tested positive on QFT. This suggests that nearly one-third of patients received INH prophylaxis that may not have been strictly necessary, raising concerns about potential overuse of prophylactic treatment.

This study has several limitations inherent to its retrospective design. Data were collected from medical records, which may be subject to documentation variability and missing information. It should be considered that patients with negative TST results may have developed false negativity due to immunosuppressive therapy. The absence of a booster dose in these patients represents one of the limitations of this study. Another important limitation of this study is that the cohort exclusively consists of IBD patients treated with anti-TNF agents. Apart from a small subset of patients who received vedolizumab as a second-line therapy following anti-TNF failure (19 patients, representing 5.4% of the cohort), data on other biologic agents such as IL (interleukin) blockers or Jak inhibitors were not available. This restricts the ability to evaluate tuberculosis risk across the full spectrum of biologic therapies. While newer agents are generally associated with a lower TB risk,^27^^-^30 demonstrating their safety profile in patients previously exposed to anti-TNF therapy would offer valuable insights, particularly in TB-endemic regions. Future studies including broader biologic cohorts are warranted to address this gap.

In conclusion, this retrospective study highlights that tuberculosis infection remains a significant challenge in patients receiving anti-TNF therapy. In this study, the incidence of LTBI was 50.4%, with 92% of LTBI-positive patients receiving INH prophylaxis. The rate of active TB infection during anti-TNF treatment was 1.7% (6/349). Of these 6 patients, 2 had received INH prophylaxis, and 2 had negative LTBI test results. Consequently, clinicians should consider the risk of active TB in patients receiving anti-TNF therapy, even in the presence of negative LTBI screening results and after INH prophylaxis.

## Figures and Tables

**Table 1.** Sociodemographic and Clinical Characteristics of the Participants (n = 349)

**Characteristics**	**n (%)**
Sex Male Female	196 (56.1) 153 (43.8)
Age (years)	Mean ± SD: 42 ± 14, Median: 40 Min: 20, Max: 86
Time from diagnosis (years)	Mean ± SD: 10 ± 6, Median: 9 Min: 1, Max: 35
Diagnosis Crohn’s disease Ulcerative colitis GI Behçet’s disease Indeterminate colitis	267 (76.5) 76 (21.8) 3 (0.9) 3 (0.9)

**Table 2.** QFT Results According to Tuberculin Skin Test Wheal Size

	**QFT Positive**	**QFT Negative**	**QFT Indeterminate**
TST wheal ≥ 5 mm (n = 162), n (%)	17/56 (30.3)	37/56 (66)	2/56 (3.5)
TST wheal < 5 mm (n = 134), n (%)	7/61 (11.4)	48/61 (78.6)	6/61 (9.8)

TST, tuberculin skin test.; QFT, QuantiFERON-TB Gold test.

**Table 3.** Sociodemographic and Clinical Characteristics of the Patients Diagnosed with Tuberculosis

**Variables **	**n (%)**
Age (years)	Mean ± SD: 39 ± 16, Median: 38.5 Min: 20, Max: 66
Disease duration when TB developed (months)	Mean ± SD: 43 ± 32, Median: 54 Min: 3, Max: 72
Interval between TB diagnosis and TNF-α antagonist therapy (months)	Mean ± SD: 18.5 ± 21, Med: 7 Min: 2, Max: 48
Diagnosis Crohn’s disease Ulcerative colitis	5 (83.3) 1 (16.7)
Drugs used when diagnosed with TB CS AZA 5-ASA	1 (16.7) 5 (83.3) 2 (33.3)
Previous medical treatment CS AZA 5-ASA	2 (33.3) 6 (100) 5 (80.3)
The treatment when TST was done CS AZA 5-ASA	4 (66.6) 4 (66.6) 3 (50)
TNF-alpha antagonist agent IFX* ADA ADA after IFX*	4 (66.6) 3 (50) 1 (16.7)
Receiving INH prophylaxis	2 (33.3)
Mean duration of INH prophylaxis	Mean ± SD: 7.5 ± 2.1, Median: 7.5 Min: 6, Max: 9
TST wheal size (n = 5) TST wheal = 0 mm TST wheal ≥ 5 mm	3 (60) 2 (40)
QFT Negative Indeterminate	4 (80) 1 (20)
TB exclusion by imaging before TNF-α antagonist initiation**	5 (83.7)

5-ASA, aminosalicylic acid; ADA, adalimumab; AZA, azathioprine; CS, corticosteroid; IFX, infliximab; INH, isoniazid; TB, tuberculosis; TNF, tumor necrosis factor; TST, tuberculin skin test; SD, standard deviation; QFT, QuantiFERON-TB Gold test.

*One patient was switched to adalimumab treatment after infliximab.

**Three patients had postero-anterior chest radiography, 1 patient had computed chest tomography, 1 patient had postero-anterior chest radiography and computed tomography. In 1 patient, data on imaging could not be obtained.

**Table 4.** Clinical Features of Patients with Tuberculosis Infection

	**Diagnosis**	**Treatment When Diagnosed with TB**	**INH Prophylaxis** ** (mo)**	**TST Wheal Size** **(mm)**	**QFT**	**The Treatment When TST Was Done**	**Chest Imaging Before Anti-TNF Treatment**	**Time from TNF-Alpha Antagonist Initiation to TB** **(mo)**	**TB** **Involvement Area**	**TB** **Exposure**	**TB** **History**	**TB** **Treatment** **(mo)**
1	UC	AZA, CS 5-ASA, ADA	No	0	Negative	AZA 5-ASA	Normal*	7	Lung + peritoneum + spleen	Yes	No	9
2	CD	AZA,ADA	9	6	Negative	AZA 5-ASA	Normal*	48	Lung	No	No	6
3	CD	5-ASA,IFX	No	0	Negative	AZA 5-ASA	Normal*	44	Lung	No	No	9
4	CD	AZA,IFX	No	NA	Indeterminate	CS	Normal**	2	Lung	No	No	12
5	CD	AZA,IFX	No	0	NA	CS AZA	Normal***	7	Lung + lymph node	No	No	9
6	CD	AZA,ADA	6	9	NA	No treatment	No data	3	Lung + spleen	Yes	No	7

5-ASA, aminosalicylic acid; ADA, adalimumab; AZA, azathioprine; CD, Crohn’s disease; CS, corticosteroid; IFX, infliximab; INH, isoniazid; TB, tuberculosis; TNF, tumor necrosis factor; TST, tuberculin skin test; UC, ulcerative colitis.; NA, Not avaible; QFT, QuantiFERON-TB Gold test.

*Postero-anterior chest radiography.

**Computed chest tomography.

***Postero-anterior chest radiography and computed chest tomography.

**Table 5.** Comparison of Patients’ Status of Being Diagnosed with Tuberculosis According to Some Sociodemographic and Clinical Characteristics

**Variables**	**TB diagnosis**		***X* ^2^**	*P* **
	**Positive (+)** n (%)*	**Negative (−)** n (%)*		
Crohn’s disease No Yes	1 (1.2) 5 (1.9)	81 (98.8) 262 (98.1)	0.2	1.0
Ulcerative colitis No Yes	5 (1.8) 1 (1.3)	268 (98.2) 75 (98.7)	0.1	1.0
GI Behçet’s disease No Yes	6 (1.7) 0	340 (98.3) 3 (100)	<0.1	1.0
Indetermine colitis No Yes	6 (1.7) 0	340 (98.3) 3 (100)	<0.1	1.0
TST wheal size TST = 0 mm 0 < TST < 5 mm TST ≥ 5 mm	3 (2.6) 0 2 (1.3)	111 (97.4) 13 (100) 155 (98.7)	–	–
QFT Negative Positive	4 (3.9) 0	99 (96) 31 (100)	1.2	0.57
INH using time (mo) <9 ≥9	1 (4.5) 1 (0.8)	21 (95.5) 125 (99.2)	2.0	0.28
INH treatment Not given Given	4 (3.2) 2 (1)	122 (96.8) 206 (99)	2.2	0.20

INH, isoniazid; TB, tuberculosis; TST, tuberculin skin test; QFT, QuantiFERON-TB Gold test; GI, Gastrointestinal

*Percent of rows.

**Chi-square test.
